# Pathologic crossroads: cardio–vascular diseases, periodontal diseases and calcium antagonists

**Published:** 2011-02-25

**Authors:** H Balan, E Popescu, G Angelescu

**Affiliations:** ‘Carol Davila’ University of Medicine and Pharmacy, Bucharest; Medical Clinic of the Clinical Emergency Hospital Ilfov County, Bucharest, BucharestRomania

**Keywords:** cardiovascular diseases, periodontal diseases, atherosclerosis, gingival overgrowth, calcium antagonists

## Abstract

**Rationale**During the last decennium t a more focused attention has been directed to the presence of chronic inflammation in cardiovascular diseases (CVD), but mainly to the high impact that this one has in generating and fastening the atherosclerotic process.

**Objective**To highlight the causal relationship between periodontal diseases (PD) and CVD. One of the most important chronic inflammations, present in the modern societies in the vast majority of the population, is represented by the periodontal diseases (PD). Both types of diseases are characterized by a high and continuously increasing prevalence. It is now clear that they share some common risk factors, but it would be of great interest, not only for a scientific purpose, but also from a possible health benefit, as PD can be prevented and treated efficiently, to prove that there is a causal link between these two pathologies.

**Methods**We will present a review of the actual data concerning their relationship

**Discussion**The study of this causal relationship is made more difficult due to the increased utilization, due to the guides' recommendations of the calcium antagonists (CA) in treating CVD.

Abreviations: AE = adverse effect; AMI = acute myocardial infarction; CA = calcium antagonists; CHD = coronary heart disease; COI = chronic oral infection; CsA = cyclosporine A; CVD = cardio-vascular diseases; DM = diabetes mellitus; DNA = deoxyribonucleic acid; GH = gingival hypertrophy; GO = gingival overgrowth; hs-CRP = high-sensitivity C reactive protein; IL = interleukine; ICAM = intracellular adhesion molecule; JE/MCP–1 = macrophage chemotactic protein; LPS– lipopolisaccharides; MIP–2 = macrophage inflammatory protein–2; MMP = matrix metalloproteinases; mRNA = messenger–ribonucleic acid; NOS = nitric oxide synthase; PAI–1 = plasminogen activator inhibitor–1; PD = periodontal diseases; TIMP = tissue inhibitor of metalloproteinase; TNF = tumor necrosis factor; USA = United States of America; UFC = units forming cultures; WHO= World Health Organization.

## Epidemiologic links: PD–CVD

The epidemiological link that exists between chronic oral infections (COI) and CVD has been largely demonstrated in the last 10 years. PDs are, undoubtedly, the most frequent type of COI [1–5].

The highest prevalence of PD and CVD is encountered in third age populations. During the last years calcium antagonists (CA) reached the class I, a level of evidence recommendation in treating coronary heart disease (CHD), the most fearful and most frequent expression of atherosclerosis. It is well known that cardiovascular disease is the single most common cause of death in the developed world and accounts for almost one million fatalities each year in United States alone [3]. Nearly half of these deaths result from coronary artery disease, another 20% from stroke [3].

So, the problem became more complicated: first to demonstrate a causal link between PD and CVD and, then, to find if CA, although they have a benefic effect in CHD, could have a deleterious effect on the periodontium.

We have already mentioned many epidemiological proofs concerning PD and CVD [1–5].

In order to prove a causative association between the two types of pathologies it would be necessary to do that by properly designed trials.

However, using hard endpoints in this kind of trials would mean impressive costs and a very long duration. That is why, maybe the surrogate end–points and some surrogate variables that are considered to have a significant predictable value for future cardio–vascular events: high sensitivity C-reactive protein (hs–CRP), homocysteine (although during the last 2 years their predictive value for future cardio–vascular events have been doubted), plasma fibrinogen, plasminogen activator inhibitor–1 (PAI–1), D–dimers, lipoproteins [1,2].

The importance of this kind of trials, that have been already done, and that we will mention is underlined by the fact that they showed not only a significant association, but also a significant pathogenic, causative relationship between PD and CVD [3,4,5].

In the last assembly of the World Health Organization (WHO) it has been strongly advocated for a good oral health, for integrating the prevention and health promotion policies between oral and general health [6].

Just a year ago a workshop regarding the connections between PD and CVD was held in Madrid (Spain) [67].

We should also mention that good preventive health care is associated with many other benefits, of which the most important can be considered the quality of life (QOL). Also, in USA General Surgeon: ‘Oral health in America’ is underlining the fact that oral health represents a fundamental part of the general health [8]. 

### Chronic infection: the main pathogenic connection

Why is it so important to study PD? Because: they have in common a predominant infectious/inflammatory origin; they are very frequent diseases: between 20 and 50% of a general population have this kind of diseases; more than 85% in the third age population (in some developed countries)[9]; if we could demonstrate that PD represent an important and reversible risk factor for CVD, the importance of preventing and treating them would be tremendous, due to the fact that the significantly important and real reversible risk factors for CVD are not so many (and those already known would imply important changes in lifestyle, that not so many subjects would agree).

PD are considered to be of multiple etiologies: the bacterial accumulation and their organization in the dental biological film represent the ‘ignition’ phenomenon; the devastating factor, regarding the destruction of the deeper periodontal tissue (by the action of the large number of leukocytes that are accumulating in the gingival tissues) is considered the host–mediated cell–mediated immune response, mediated by large amounts of inflammatory mediators: cytokines–chemokines and matrix metalloproteinases (MMP)[10,11,12].

We must underline that MMP are involved in the immune pathogenic process of PD; they also include collagenase (that represents a reliable marker of the active disease when the plasmatic and salivary concentrations are significant). We could also use as a clear–cut edge a high concentration of collagenase, to make the difference between a simple gingival inflammation (gingivitis) and a very severe, with destructive potential PD.

PD have the following risk factors: increasing age, specific periodontal pathogens: *Porphyromonas gingivalis, Tanerella forsythia, Fusobacterium nucleatum*, some ethnic minorities, low socio–economic status, male gender, stress, increased body weight, dyslipidaemia, hypertension, diabetes mellitus (DM), cigarette smokers, the last five common cardio–metabolic risk factors being associated with increased odds of prevalence of periodontitis. Important risk factors for PD are considered: poor oral hygiene; diabetes mellitus; immune depression.

Concerning the risk factors of CVD, these ones have been classified as: **conventional risk factors**: smoking, hypertension, dyslipidaemia, insulin resistance and diabetes mellitus, lack of exercise, obesity; **novel atherothrombotic risk factors**: high sensitivity C–reactive protein (hs–CRP) and other markers of inflammation, homocysteine and lipoprotein (a); **thrombotic markers of risk**: fibrinogen, D–dimers, abnormalities in intrinsic fibrinolysis. All these risk factors can have a common pathway that is represented by **endothelial dysfunction and inflammation**.

In considering their possible causal relationship, we must start with some wrong beliefs, very common even in medical populations: an action that even a scientific authority as the American Academy of Periodontolgy can take: ‘Facts and fallacies about periodontal diseases’:

- it is natural that dental loss appears with age increase: false–PD represent the main cause for edentations appearing before 35 years old;
- the main cause for dental loss are cavities: false–in 1996 American Dental Association (Colgate survey) has demonstrated a 2:1 ratio in favor of PD;
- those with PD generally have a poor hygiene: false–up to 30% of the general population is genetically susceptible of PD (they have a 6 times greater tendency for PD);
- the oral health does not affect general health: false–PD are associated with: CVD, ‘small for date’ newborns, prematurity, stroke, diabetes mellitus.
- mucosal infection is a rather small, minor one: false–the mass of the tissue in the oral cavity equals the skin of the arms, from elbow to wrist;
- bleeding of the gums is a natural phenomenon: false–bleeding is one of the most frequent signs of gingival disease;
- gingival treatment is painful: false;
- gingival diseases are easy to recognize, so that any dentist could see very reliable all the abnormalities;
- because gingival infections are generated by microbial agents, one can use antibiotics for their treatment: false–that create the great risk of selecting multi-resistant species;
- pregnant women should skip their periodic dental controls, because it's well known that their increase in dental mobility is only temporary: false–the diminished immunity during this period make mandatory a periodical periodontal control.

Let us now return to the initiating process: anytime the local blood flow decreases, a common fact for both CVD and PD, a complex array of metabolic abnormalities results from an insufficient delivery of oxygen and nutrients combined with a diminished removal of waste products. The oxygen deprivation is a trigger for the activation of the clotting system; the tissue factor pathway represents the main trigger for the pro–coagulant mechanism.

The normal endothelium is characterized by reduced, if any, presence of tissue factor, a fact that is necessary in order to maintain a normal blood flow.

Oxygen deprivation is generating a rapid increase in tissue factor expression in the vessel wall, due to the rapid increase in tissue factor expression (by a prompt increase in the level of mRNA tissue factor). The hypoxia–induced expression of the gene is closely linked to a tissue factor promoter, this phenomenon being followed by: an increase of vascular permeability; a pro–coagulant significant effect; an up–regulation of the cell adherence, of ICAM molecules and of the inflammatory mediators associated with leukocyte migration and activation, (**the leukocytes have an important role in tissue damage, consequent to ischemia**).

Similarly, PAI–1, the cytokine interleukin (IL)–1beta and chemokines macrophage inflammatory protein (MIP)–2 and macrophage chemotactic protein (JE/MCP)–1 are up regulated by ischemia (all these molecules being linked to leukocyte–mediated tissue damage).

The host–tissue response is characterized by a local accumulation of neutrophils, macrophages, different lymphoid cells, that will elicit, together with the adjacent host–tissue cells, an immune–inflammatory response, characterized by the release of different cytokines and prostanoids: IL–1, IL–6, IL–8, TNF–alpha, prostaglandin E2, different MMPs (these ones playing a crucial role in the destruction of the bone and of the connective tissue).

Together with the fact that lipopolysaccharides (LPS) and other products of the Gram negative bacteria may stimulate, cytokine production; hypercoagulability; monocyte activation; liver activation, that will all have a direct or/and indirect effect on the vascular walls, generating **endothelial dysfunction**.

Therefore, the mechanisms by which periodontal bacteria may contribute to CVD: blood platelet aggregation; enhanced low–density lipoproteins and cholesterol deposition in the arterial wall; direct invasion of the cardiac and vascular endothelium [13,14,15].

Regarding the different components of the immune process implied by the presence of PD, it is important to underline the following:MMP–8 can be defined as a collagenase found in the gingival tissue, with high values in the crevicular fluid during periodontal diseases. It is considered, together with IL–1beta salivary level, a reliable marker of PD. MMP–8 is the only proteinase that has the capacity of collagen I and III fragmentation, the main mechanism for the periodontal tissue destruction, so high levels of MMP–8 are correlated with severe PD. A 3 times greater than normal concentration of MMP–8 can be used as diagnostic tool to differentiate a severe form of PD from a simple inflammation (gingivitis).

An efficient periodontal treatment is followed by a normalization of these values [6]. More severe is the PD, higher are the values of MMP–1, MMP–2, MMP–3, MMP–8 and MMP–13 [10,11].

**All the metalloproteinases play an important role in the immune response and in atherogenesis**

Elevated levels of the extra–cellular matrix metalloproteinase inductor and of the tissue inhibitor of metalloproteinase (TIMP) in the gingival crevicular fluid participate in PD progression [6].

**The periodontal microorganisms are generating the PD and are stimulating the host immune response**

The first step of the answer to periodontal microorganisms is represented by an infiltrate of mononuclear cells in the gingival connective tissue.

The second step is represented by the release, from these ‘recruited’ macrophages and lymphocytes of pro–inflammatory cytokines and growth factors, as IL–1beta, TNF–alpha, enabling MMP gene transcription.

The fibroblasts, keratinocytes, macrophages, endothelial cells respond to catabolic products from the regulating MMP expression.

The osteoblasts, activated by the same mechanism, induce bone desorption by fragmentation of the extracellular matrix constituents. An up–regulation of the cells adherence, ICAM molecules and of the mediators of inflammation is associated with leukocyte migration and activation.

Given the large number of receptors, cofactors and mediators involved in vascular pathologies, it is important to understand where they are positioned in the host response cascades in which they participate.

Although the bacteria are the main etiological agents, most of the tissue destruction occurs because of the host immuno–inflammatory response against the microbial challenge. This response is modulated by both genetic and environmental risk factors; there are evidences from twin studies that genetic factors explained a substantial proportion (almost 59%) of the variation in the severity and extent of periodontitis [16, 17]. 

**Inflammation is the initiating process of the atheroma formation and chronic infections including PD might influence systemic or vascular inflammation processes. **

This may also help understanding the complex interplay between recognized (i.e. increased age, male gender, dyslipidaemia, obesity,  cigarettes smoking, DM, etc.) and novel cardiovascular risk factors, chronic infections and their associated inflammatory processes that can directly influence the physiopathology of atherosclerosis and thus alter CVD risk.

The source of bacterial pathogens: caries and PDs, derives from the adhered bacterial populations to the tooth surfaces (biological films with a bacterial density of about 10^11^ UFC/mg). The bacterial communities present and adherent to the tooth surfaces are among the most complex existing in nature, due to the very complex ecosystems in the oral cavity, and especially in the periodontal environment, where a very dynamic co–existence between pathogenic and saprophyte bacteria exists, on one side, and on the other side the host cell defences [18]. Patients with PD have a periodontal pocket that means an area especially prone for bacterial dissemination into the systemic circulation. Transient bacteriaemia has been reported after different preventive and therapeutic dental procedures: after scaling and root planning, after periodontal surgery, and dental extractions [19]. In addition, recent studies have suggested that everyday/common actions, such as chewing and tooth brushing contribute more significantly to the cumulative exposure of the vascular system to the oral bacteria [20]. Although most of the bacteriaemias are transient, it has long been recognized that bacteria in the blood stream may cause distant site infections. 

Several studies have demonstrated the presence of certain oral bacteria in atherosclerotic plaques and abdominal aortic aneurysms, in particular species implicated in the pathogenesis of periodontitis [21–23]. 

The most common signs of this rather lack of symptoms condition are: gingival bleeding and swelling; these other ones represent the hallmark of the more advanced forms: gingival recession, drifting and mobility of the teeth, suppuration and are due to the progressive destruction of the dental supporting tissues, which finally leads to alveolar bone destruction up to the apex of the tooth, and even to the tooth exfoliation.

The PD are defined by the anatomic destruction of the tissues that are sustaining the teeth, that appears after a pathological process.

By using this definition, the severity is measured by the amplitude of the destruction. However, using tissue destruction as definition for PD is a way to look only to consequences, but no to the pathological process itself.

Considering that PD are chronic infections that will lead to a systemic inflammation, they could be, and it would be a more scientific way of considering them, alternatively by the infectious burden or by their systemic inflammatory consequences. Of course, it is very clear that there is a direct relation between the amplitude of the destruction and the magnitude of the systemic manifestations of infection.

When we are dealing with important tissue destruction and the presence of a periodontal pouch, there is a clear association with many markers of inflammation: hs–C protein, homocysteine, fibrinogen, PAI–1, D–dimers, proinflammatory cytokines plasmatic concentration [1,2,21–26].

This inflammatory process represents the result of the interaction between pathological agents and the reactivity of the host [27–31].

For the beginning of a productive inflammatory process the pathogenic agents must act as an irritating factor, so that the periodontal marginal tissue reacts by a proliferative type of inflammation; in such type of inflammation the main process is represented by the reaction of the local tissue components that react by: hyperplasia, metamorphosis and mobilization, 3 stages in the defensive–adaptive process.

The important changes in the periodontal chorion have an impact on the cement and alveolar bone.  

For the aim of this review, it is very important to underline that we are dealing with a chronic infection, leading to systemic inflammation. Considering PD this way, we could alternatively define them by the infection load or by the systemic inflammatory consequences, thus dealing more with the pathophysiological process than with the secondary destruction [32], investigating 657 subjects: the following microorganisms have been proved to be present in the dental film and in atherosclerotic lesion: *Actinobacillus actinomycetem comitans, Porphyromonas gingivalis, Tannerella forsythia, Treponema denticola*. It has also been proved that they are connected with intima–media thickness ratio and with hs–CRP levels. 

Although the dental plaque is by far the most common cause for gingivitis, many other factors can contribute to worsening the condition: **drugs**: antidepressants, those used for flu symptomatic treatment–they are decreasing the salivary secretion (generating xerostomia and severely decreasing the auto–cleaning capacity of the oral cavity); antiepileptic medication, **calcium antagonists (CA)**, immune–depressing drugs (Cyclosporine A– Cs A); erithromycine, claritromycine, metronidazole, ketoconazole; **viral and fungal infections; other diseases/conditions**: pemphigus, pemphigoid; hormonal disturbances/ modifications (pregnancy, as an example); **deficient nutrition**: in calcium, C and B vitamins.

### Benefits and adverse effects of CA

Old people commonly have many co–morbidities, each of them requiring one or more drugs [33]. Adverse reactions are more frequent in elderly population, due to a variety of reasons, but most frequently due to polipragmasia: the risk is 6% for 1 drug; 50% for 5 drugs; 100% for those taking more than 8 drugs. Moreover, this can generate other problems: the medical conditions that deserve medication; the effect of these conditions/medications on the healing capacity; the impact of these medications on the usual treatment schemes of the dentist.

Calcium antagonists (CA) exert their effect by inhibiting the Ca^++^ influx by the specialized channels, real pharmacologic receptors.

Their increasing use is explained by the benefic effects they have: to inhibit the contraction of the myocardial fibers; to depress the excitation generating the slow potentials; to relax smooth muscle fibers, especially the vascular ones, an effect that explains their first successfully utilization: in treating hypertension; but, nowadays they are increasingly used in treating CHD (class I recommendation, a level of evidence) where, by influencing the inward calcium current they can diminish platelet activation, the vascular contraction, the activity of endothelial cells, the monocytes invasion, the ‘foam cells’ appearance, so that they can reduce the development of new atherosclerotic lesions.

All the following are benefic actions in treating CHD: the direct and benefic effect on the endothelial function; the prevention of the noxious action of the endothelial mediators; to act as substitutes for the deficient endothelial factors; offers vascular protection by increasing NO–formation in the endothelial cells, ameliorating endothelial function.

In stable angina, AC are: diminishing the effort–induced vasoconstriction; reducing post–load by reducing blood pressure; diminishing the increase in cardiac frequency effort–induced and diltiazem and verapamil have a negative inotropic effect.

Nifedipine was the first representative of the class of drugs that proved to have a protective effect for the cardiac cells from apoptosis, after acute myocardial infarction (AMI). The most frequent adverse effect (AE) that is associated with AC is represented by gingival overgrowth (GO), or after different authors gingival hypertrophy (GH).

Gingival hyperplasia is a wrong denomination, since it's not an increased number of cells, but an increase of the volume of the extra–cellular tissue. We prefer to use the term of GO because the volumetric increase of the gingival tissue is due to an increase of the amount of extra–cellular gingival macromolecules and the appearance of a great number of inflammatory cells.

Inflammation of the gingival tissue from the bacterial plaque and the subsequent development of gingival crevicular fluid may allow sequestration of the CA, thus predisposing the tissue to a localized, toxic effect and to the development of GO.

GO can be observed after a few weeks or after a few years from the beginning of the treatment, being reversible after interruption of the drug that is generating her, a fact that underlines the necessity of periodic, specialized examinations, because **the real definition of GO is represented by the increase, with more than 1 mm of the inter–dental papilla (even in an isolated space)**.

The overgrown tissue has a lobulated /granular aspect.

GO is aggravated by gingival inflammation and by a poor oral hygiene.

We must mention that oral surgeons have made the first observations about this AE of CA [34,35].

For a definite diagnostic it is mandatory to include all the following: medical and dental history, including a summary of medical (drug) therapy; detailed examination of the teeth, of the periodontium, of the head and of the neck; photographs of the GO; dental X–ray; different blood tests; tissue biopsies or/and gingivectomy [36].

The number of fibroblasts (together considered: dead or functional) obtained from a normal gingival tissue has increased, the cellular death being inhibited by nifedipine, similar effect, concentration–dependent being obtained for: nicardipine, diltiazem, verapamil.

Fibroblasts from nifedipine–induced fibrotic gingival hyperplastic epithelium, with elongated, branched rete pegs, have been characterized with respect to several cellular functions, which could contribute to the characteristic clinical overgrowth of the gingival: collagen synthesis and breakdown; glycosaminoglican production; fibronectin synthesis; proliferation.

The data suggested that nifedipine affects the metabolism of fibroblasts derived not only from gingival, but also from other collective connective tissues.

Thus, nifedipine responder cells are present in tissue other than gingival.

There was an inverse relationship between in vivo tissue levels of IL–1 beta and in vivo responsiveness to nifedipine of fibroblasts derived from that tissue.

Nifedipine–induced overgrowth of connective tissues, other than gingival, probably does not occur because of the reliability slow rate of collagenous protein synthesis by resident fibroblasts or because of alterations in collagen deposition / desorption within susceptible tissues produced by nifedipine on collagenase synthesis.

The gingival epithelium is para–keratinized and exhibit elongated rete pegs. The underlying connective tissue comprised dense collagen fibers and the inflammatory cells, which were present in the connective tissue, were mainly plasma cells and lymphocytes.

Nitric oxide synthase (NOS) induction has been completely prevented by incubation with nifedipine and the cellular death induced by LPS.

Adherence and LPS–stimulated macrophage–induced death of fibroblasts can be the mechanism of GO during chronic treatment with CA [37].

GO induced by nifedipine is characterized by an accumulation of collagen components in the gingival connective tissue and epithelial hyperplasia with prolonged and branched rete pegs, that are penetrating in the connective tissue, the epithelial layer being thickened, acantotic, parakeratozic.

Nifedipine, as the majority of CA induces alterations of the intracellular level of calcium in the gingival cells and can induce local inflammatory factors that are taking the place of collagen of the connective tissue, thus generating GO.

CA have functional connections: nutritional synergy with folic acid, L–carnitine, different supplements that are diminishing the performance of the drugs: vitamin D, calcium, magnesium

GO can determine **speech difficulties, mastication difficulties, dental eruption difficulties, esthetic disturbances**.

The marginal gingival tissue begins to have an excessive growth, especially at the anterior margin and could finally completely cover the occlusal surface of the teeth, creating major chewing problems.

By electron microscopy, one can see the disappearance of the cellular bonds, the cellular exfoliation, and the fact that the normal honeycomb structure formed by the pits is disturbed [38,39].

Other gingival surfaces presented many round, oval or dome-like shapes that were replacing some micro–bridges: parallel, reticular, having the shape of some fingerprints.

The terminal differentiation of epithelial keratinocytes has been proposed as a specialized form of programmed cellular death (apoptosis).

A loss of cellular adherence does not have as a result the beginning of apoptotic destruction of DNA, but an increased sensitivity of the cells to this fact, that was triggered by calcium [38,39].

This susceptibility appeared in parallel with the **irreversible loss** of the growing potential, volucrine accumulation (they are becoming competent for the apoptosis induced by calcium).

Nifedipine determined an increase of the number of lymphocytes, and their number diminished after periodontal specialized treatment.

Nifedipine determined GO in 5–39% (40–46) of subjects, the main factors influencing GO are the duration of the drug treatment (more than 4 years); the presence of the gingival inflammation; the severity of the gingival inflammation (the greatest inflammatory scores); male gender.

Those with the highest inflammatory scores and those who had a longer than 4 years duration of the treatment, had an increased tendency for a higher incidence and severity of GO.

Nifedipine increased the number of cells by increasing cellular proliferation; increased timeline uptake; increased the protein synthesis as a marker, the glucosamine uptake); increased the viability of the cells (as a marker, the LDH liberation). The results of long duration treatments showed an inhibition of proteoglicans synthesis.

GO was more significantly present: in the inter-dental papilla of the oral anterior region; the tissue is firm and pale, with a normally stippled pattern, and with a histopatologic aspect: a conspicuous increase of fibrous connective tissue; an inflammatory infiltrate; the presence of the hyperplasia of the underlying epithelium.

It has been suggested that the fine regulated balance between extra–cellular matrix synthesis and degradation may be disrupted, resulting in an accumulation of excess connective tissue components within the gingival tissue.

All the studies [48–52] showed a directly proportional relationship between the degree of inflammation and apoptosis; a strong inversely proportional trend between the degree of inflammation and the numbers of cells undergoing mitosis.

These data suggest that epithelial cell proliferation and viability are inversely associated with the degree of gingival inflammation, once a putative ‘adaptative threshold’ is exceeded.

Fibroblasts derived from the overgrown tissue exhibit a level of protein synthetic activity approximately twice that of comparable cells obtained from controls: 20% of the protein synthesized by cells from the affected tissue is collagen (only about 11% of the protein produced by control cells).

CA appear to induce or select fibroblasts characterized by enhanced levels of protein synthesis and collagen production. This alteration persists through several cell replications in vitro in the absence of drug.

Addition of the drug resulted in reduced levels of collagenolytic activity in the culture medium

MMP–1 mRNA expression was significantly reduced in overgrown compared to normal tissue. GO may be explained by a CA–induced inhibition of collagenolytic activity within the gingival tissues. The fibroblasts and collagen density increased in parallel with the severity of the overgrowth.

Apoptosis plays a critical role in the regulation of inflammation and the host immune response; CA determined statistical significant increase in keratinocyte apoptosis; significantly higher gingival index; significantly higher overgrowth scores; significantly more probing sites greater than 3 mm.

A very solid proof about a causal connection between PD and CVD is the direct relation between the number of teeth still present in third age subjects and respectively the average life expectancy [53].

In the scientific notifications (included in every box of drugs) are mentioned only those AE with such a severity that they determined the therapy interruption.

We must mention, as an example, that GO induced by nifedipine is mentioned as being present in 0,5 % to 83% in those treated (Westside Veterans Administration); it worth underline the fact that the high percentages were obtained by periodontologists. The important modifications already presented, generated by CA can have a very important impact on the periodontium, on the cement and, respectively on the alveolar bone. 

When a significant destruction and a periodontal pouch is present, there is a clear association with many inflammatory biomarkers, as hs–CRP, homocysteine, fibrinogenemia, PAI–1, D–dimers, Lps  or the pro–inflammatory cytokines that are increased and can be determined at plasmatic and local level. All these pathologic changes are part of a productive inflammatory process and represent a result of the interaction between the pathogen agents and the reactivity of the entire body [54–60]. Epithelial hyperplasia that develops not only in surface, but also profoundly, can generate epithelial islands inside the chorion, by this fact explaining the disappearance of contact inhibition, formatting, in this kind of cases lesions that are close to the dystrophic–inflammatory processes and the degenerative ones.

The important modifications already presented, generated by CA can have a very important impact on the periodontium, on the cement and on the alveolar bone. 

The precise search and diagnosing GO, is not always optimal. That's why it's necessary to underline, once again, the importance of the periodontal check (best option: monthly), made by specialists, beginning a few weeks after the initiation of the therapy with CA, till after more than 4 years (the period of maximal incidence of GO), for offering only the benefits that CA can have in CVD.
